# Adherence and Reactogenicity to Vaccines against SARS-COV-2 in 285 Patients with Neuropathy: A Multicentric Study

**DOI:** 10.3390/brainsci12101396

**Published:** 2022-10-16

**Authors:** Salvatore Iacono, Vincenzo Di Stefano, Paolo Alonge, Claudia Vinciguerra, Giammarco Milella, Francesca Caputo, Piergiorgio Lasorella, Gabriele Neto, Antonia Pignolo, Angelo Torrente, Antonino Lupica, Paola Ajdinaj, Alberto Firenze, Stefano Tozza, Fiore Manganelli, Antonio Di Muzio, Giuseppe Piscosquito, Filippo Brighina

**Affiliations:** 1 Neurology Unit, Department of Biomedicine, Neuroscience, and Advanced Diagnostics (BiND), University of Palermo, 90129 Palermo, Italy; 2 Neurology Unit, University Hospital “San Giovanni di Dio e Ruggi D’Aragona”, 84131 Salerno, Italy; 3 Neurology Unit, Department of Basic Medical Sciences, Neurosciences and Sense Organs, University of Bari “Aldo Moro”, 70124 Bari, Italy; 4 Department of Neurology, SS Annunziata Hospital, 66100 Chieti, Italy; 5 Department of Health Promotion, Mother and Child Care, Internal Medicine and Medical Speialities, University of Palermo, 90127 Palermo, Italy; 6 Department of Neuroscience, Reproductive and Odontostomatology Science, University of Naples Federico II, 80131 Napoli, Italy

**Keywords:** SARS-CoV-2 infection, COVID-19 vaccines, reactogenicity, vaccine safety, vaccine hesitancy, neuropathy, autoimmune neuropathy, hereditary neuropathy, CIDP

## Abstract

Background: The safety of the new vaccines against SARS-CoV-2 have already been shown, although data on patients with polyneuropathy are still lacking. The aim of this study is to evaluate the adherence to SARS-CoV-2 vaccination, as well as the reactogenicity to those vaccines in patients affected by neuropathy. Methods: A multicentric and web-based cross-sectional survey was conducted among patients affected by neuropathy from part of South Italy. Results: Out of 285 responders, *n* = 268 were included in the final analysis and *n* = 258 of them (96.3%) were fully vaccinated. Adherence to vaccination was higher in patients with hereditary neuropathies compared to others, while it was lower in patients with anti-MAG neuropathy (all *p* < 0.05). The overall prevalence of adverse events (AEs) was 61.2% and its occurrence was not associated with neuropathy type. Being female and of younger age were factors associated with higher risk of AEs, while having an inflammatory neuropathy and steroids assumption were associated with a lower risk (all *p* < 0.05). Younger age, having had an AE, and COVID-19 before vaccination were factors associated with symptoms worsening after vaccination (all *p* < 0.05). (4) Conclusions: Patients with neuropathy showed a high level of adherence to COVID-19 vaccination. Safety of vaccines in patients with neuropathies was comparable to the general population and it was more favorable in those with inflammatory neuropathy.

## 1. Introduction

### 1.1. COVID-19 and Neurological Disorders

The novel “Severe Acute Respiratory Syndrome Coronavirus 2” (SARS-CoV-2) is a novel respiratory coronavirus leading to coronavirus disease 2019 (COVID-19). COVID-19 usually leads to fever, cough, and dyspnea, but it might later progress to life-threatening conditions, such as lung injury and multiple organ failure [1,2]. Since SARS-CoV-2 showed marked neurotropism, several neurologic manifestations are emerging; also, the infection may be detrimental in patients with pre-existent neurological disability [3,4]. Patients with neuromuscular disease (NMD) might have an increased risk of severe COVID-19; thereby, they are considered vulnerable and scientific task forces have provided recommendations for the care of these patients [5,6,7,8]. However, social restrictions have significantly changed the management of NMD patients with troubles in accessing treatments and physiotherapy; also, the detrimental effects of the confinement measures on quality of life of the NMD patients has been shown by recent studies [8,9].

### 1.2. Vaccine Hesitancy (VH)

Vaccination against COVID-19 represents an essential medical strategy to protect vulnerable patients to achieve herd immunity, thus restoring normal life after the outbreak of COVID-19. To date, two nucleoside-modified mRNA (i.e., BNT162b2 and mRNA-1273) and two viral-vectors-based vaccines (i.e., ChAdOx1nCov-19 and COVID-19 Vaccine Jansen) are approved by European Medicines Agency [10]. The safety and the efficacy of these vaccines were established through randomized controlled trials (RCTs), despite many people still being doubtful about the safety of vaccination, with different rates of vaccination hesitancy (VH) among the population [11,12,13]. Recent recommendations encourage vaccination against COVID-19 in patients with neuropathy, although these are based on previous data about the safety of other vaccines in dysimmune neuropathies and other autoimmune disease [14]. The lack of post-marketing studies exploring the safety of vaccines against SARS-CoV-2, together with several case reports published describing the occurrence of neuropathy following vaccination, might discourage some patients from vaccination. Therefore, VH is a dangerous global health threat, and its wide diffusion might make it difficult or impossible to achieve herd immunity [12]. Unfortunately, vulnerable patients with polyneuropathy have not been included in RCTs evaluating safety and efficacy of COVID-19 vaccines; hence, further observational studies are needed in these patients. To date, there are no studies evaluating the impact of COVID-19 vaccines in patients with polyneuropathy. In this scenario, we performed a cross-sectional survey in patients with polyneuropathy attending to five different Italian Centers specialized in the care of neuromuscular rare diseases with the aims to investigate the adherence to the Italian vaccination program, as well as the safety in a real-life setting, in terms of local and systemic reactogenicity reactions to COVID-19 vaccines.

## 2. Materials and Methods

### 2.1. Study Design and Aims

We performed a multicentric web-based cross-sectional study to assess the adherence to vaccination against SARS-CoV-2 and the prevalence of adverse events (AEs) following this vaccination in patients with neuropathy. The first aim is to assess the adherence to vaccination in patients with neuropathy. Moreover, we aim to explore whether the prevalence of AEs differs depending on gender, age, type of neuropathy, COVID-19 before vaccination, and medications assumption.

### 2.2. Patient Collection

Patients with neuropathy attending the Neuromuscular Clinics of Palermo (University Hospital “Policlinico Paolo Giaccone”), Bari (University Hospital “Azienda Ospedaliera Universitaria Policlinico of Bari”), Salerno (University Hospital “San Giovanni di Dio and Ruggi D’Aragona”), Napoli (University of Naples “Federico II”), and Chieti (SS Annunziata Hospital) were invited to fill the web-based questionnaire. The invitation to participate in the survey was widespread via social media (Facebook and WhatsApp) and e-mail to patients along the period from August 2021 to June 2022. The inclusion criteria were age > 18 years at the time of the study, written consent to participate, and a definite diagnosis of neuropathy. According to diagnostic criteria, participants were divided into five groups: dysimmune, hereditary, diabetic, toxic, and deficiency neuropathies. Moreover, patients with dysimmune and hereditary neuropathies were further categorized according to specific diagnosis.

### 2.3. Data Collection

Data collection was carried out through a web-based questionnaire created in Google Forms (Google LLC, Menlo Park, CA, USA). The questionnaire was self-administered and contained two sections and 17 mandatory questions, 13 of which provided the possibility of a multiple choice, while the remaining 4 provided an open-field response. In Section 1, the questionnaire aimed to collect the signed informed consent form to participate in the study, demographic data (i.e., gender and age), and clinical features (i.e., type of neuropathy, COVID-19 or not, and its severity), while, in Section 2, vaccination information (i.e., type of vaccine administered, number of doses, occurrence of AEs following vaccination and type of AEs, and medications at the time of vaccination) and subjective evaluation of the neuropathy-related symptoms after vaccination (i.e., stable, improved, or worsened) were collected. Adverse events (AEs) refer to the transient local or systemic reactogenicity reported by patients in a window period of seven days following vaccination. The occurrence of local pain, asthenia, cephalalgia, myalgia, fever, gastrointestinal symptoms, and erythema was collected without a distinction between doses. The answers were saved by clicking on the “send” button provided at the end of the questionnaire. Finally, all the collected data were updated or confirmed for each patient at scheduled follow-up visits performed during the recruitment period. The questionnaire in its original language (Italian) and in English language is provided in Supplementary File S1.

### 2.4. Informed Consent form and Data Privacy

Participants’ responses were anonymous and confidential, in accordance with Google’s privacy policy (https://policies.google.com/privacy?hl=en) accessed on 1 September 2022. Participants were not allowed to provide any contact information, such as name, surname, phone number, or home address. The first question of the questionnaire provided the acquisition of the written informed consent form to participate in this anonymous study; also, during the informed consent acquisition process, each participant was confident that all data would be used for research purposes only. If the participant denied consent, the questionnaire ended automatically, and the response was not recorded. In addition, participants were able to stop participating in the study and leave the questionnaire at any stage before the end of the questions. If they decided to leave the study, their answers would not be saved.

### 2.5. Statistical Analysis

The distribution of the quantitative data was evaluated by using the Kolmogorov–Smirnov test. Quantitative variables are reported as mean and standard deviation (SD) or median and interquartile range within squared brackets [IQR]. Qualitative variables are presented as numbers and percentages. Associations between categorical variables were assessed by using Chi-squared and Fisher’s exact tests. Risk is reported as the odd ratio (OR) with confidence interval (CI) at 95% within squared brackets. Mann and Whitney U and Kruskal–Wallis tests were used to analyze nonparametric quantitative data. Correlations analyses were carried out by using Spearman’s test. The level of statistical significance has been set at 0.05 for all the statistics; for Chi-squared and Fisher’s exact tests, a two-tailed significance was used, while, for Spearman’s test, a one-tailed one was used. All tests were performed using SPSS (IBM Corp. Released 2019. IBM SPSS Statistics for MacOS, Version 26.0. Armonk, NY: IBM Corp). The statistical power analysis was performed by using G*Power Software for MacOS v 3.1 [15].

## 3. Results

**Figure 1:**
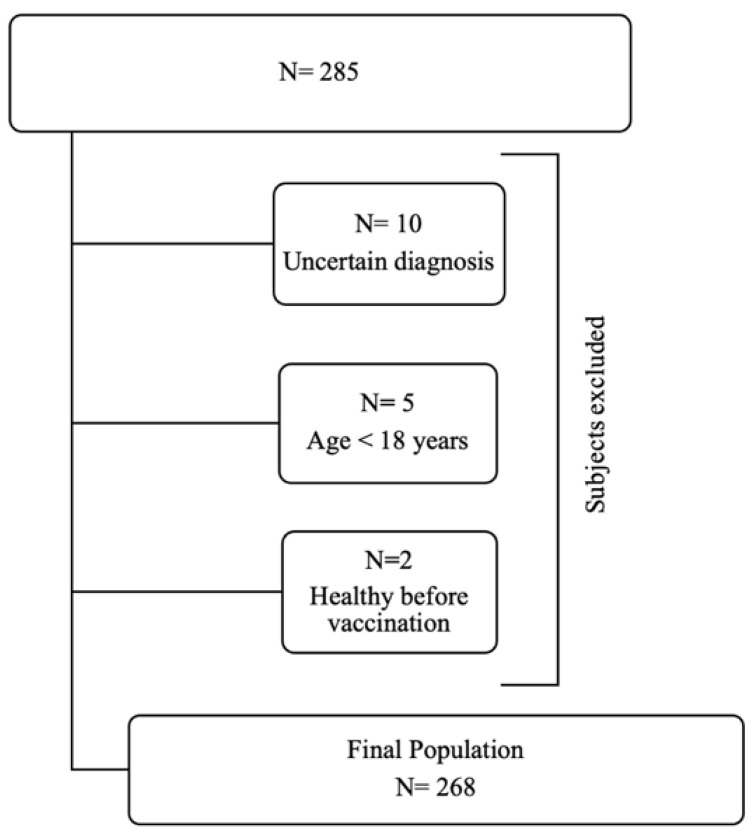
Recruitment procedure according to study’s inclusion criteria.

All the continuous variables have a not-normal distribution according to Kolmogorov–Smirnov test (all *p* < 0.05). The Kolmogorov–Smirnov and graphs derived from Spearman’s analyses are provided in Supplementary File S2. A total of 285 subjects (105 female, median age 60 years (48–68)) completed the web-based questionnaire and 17 of these were excluded (Figure 1).

**Figure 2:**
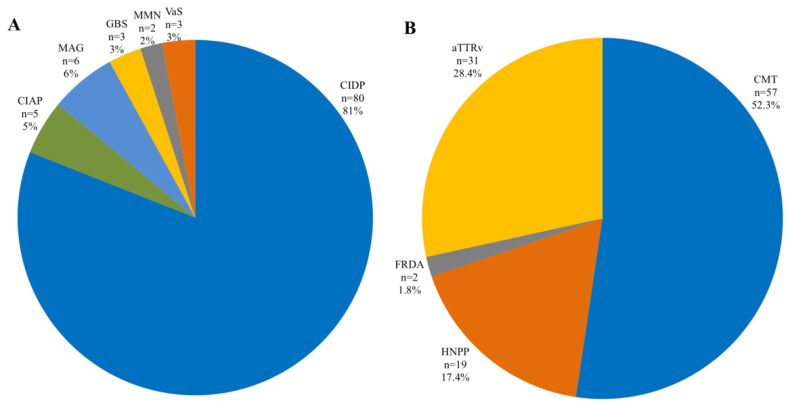
Prevalence of polyneuropathy subtype in inflammatory (**A**) and hereditary (**B**) polyneuropathies. CIDP, chronic inflammatory demyelinating polyneuropathy; CIAP, chronic inflammatory axonal polyneuropathy; MAG, myelin-associated glycoprotein; GBS, Guillain–Barré syndrome; VaS, vasculitis; ATTRv, hereditary transthyretin amyloidosis. CMT, Charcot–Marie–Tooth. HNPP, hereditary neuropathy with liability to pressure palsies; FRDA, Friedreich ataxia.

Chronic inflammatory demyelinating polyneuropathy (CIDP) and Charcot–Marie–Tooth (CMT) were the most common neuropathy subtypes (Figure 2).

From a total of *n* = 268 participants, 258 (96.3%) were fully vaccinated against SARS-CoV-2 (102 F; median age 59 years (48–68)), while *n* = 10 (3.7%) did not receive the vaccination (3 F, median age 60 years (53–68)), without difference in gender and between age groups (all *p* > 0.05). The median time between the last dose of vaccine and the compilation of the questionnaire was 151 days (103–204). The post hoc achieved power of this study (Sample size *n* = 258; Df = 1; α = 0.05) ranged from 58.8% to 95.3% based, respectively, on the lowest (phi = 0.136) and the highest (phi = 226) effect size values found after the Chi-square tests.

### 3.1. Adherence to Vaccination

Adherence to vaccination was higher in patients with hereditary neuropathy compared to other groups (χ^2^ = 7.12; phi = 0.16; *p* = 0.008; Table 1). Among the patients with inflammatory neuropathy, adherence to vaccination was comparable to other groups (Table 1), although those with anti-MAG neuropathy reported a lower adherence (*n* = 4/6, 66.7% vs. 254/262, 97%; Fisher’s exact test: *p* = 0.017). A total of 249 patients received mRNA-based vaccines (*n* = 219/249, 87.9% BNT162b2 and *n* = 30/249, 11.6%, mRNA-1273), while 9 received viral-vectors-based vaccines (*n* = 6/8, 75%, ChAdOx1nCov-19 and *n* = 2/8, 25%, COVID-19 Vaccine Jansen). At the time of the interview, *n* = 8 patients (3.1%) received only a dose of vaccine, whereas 129 (50%) received two doses and 121 patients (46.9%) received three doses. Patients who underwent viral-vector-based vaccination received fewer doses compared to those who received nucleoside-modified mRNA-based vaccines (1.78 ±0.44 vs. 2.46 ±0.54; *p* = 0.001), while no significant differences emerged comparing the number of doses received from participants according to neuropathy subtype (*p* = 0.46; Table 1).

### 3.2. Adverse Events

Out of 258 patients, a total of *n* = 158 (61.2%) experienced at least an AE following vaccination (Table 2). Local pain was the most common AE (67.7%), followed by asthenia (44.9%), myalgia (23.5%), fever (20.2%), cephalalgia (18.8%), gastrointestinal symptoms (5.7%), and erythema (1.7%), while 77 patients (48.7%) experienced at least two different AEs (Table 2).

**Figure 3:**
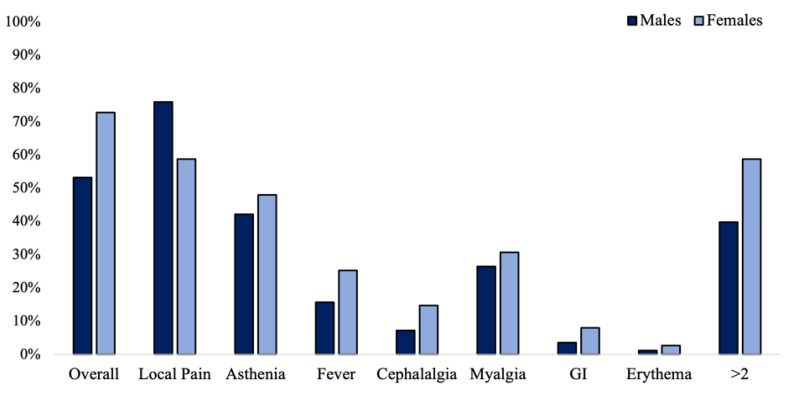
Prevalence of adverse events in *n* = 258 vaccinated patients depending on gender.

AEs were more common in females compared to males, despite the neuropathy subtype (73.5% vs. 53.2%: χ^2^ = 10.733; phi = 0.204; OR= 2.44 [95% CI: 1.42–4.2]; *p* = 0.001). Overall, females experienced more commonly at least two AEs compared to males (58.7% vs. 39.8%; χ^2^ = 5.64; phi= 0.189; OR= 2.15 [95% CI: 1.4–4]; *p* = 0.018), as well as reporting the higher number of AEs (1.88 ± 0.89 vs. 1.72 ± 1.11; *p* = 0.046). In contrast, local pain was more common in males (75.9% vs. 58.7%; χ^2^ = 5.35; phi = −0.184; OR= 2.22 [95% CI: 1.12 to 4.34]; *p* = 0.021). The prevalence of each AE according to gender is reported in Figure 3.

**Figure 4:**
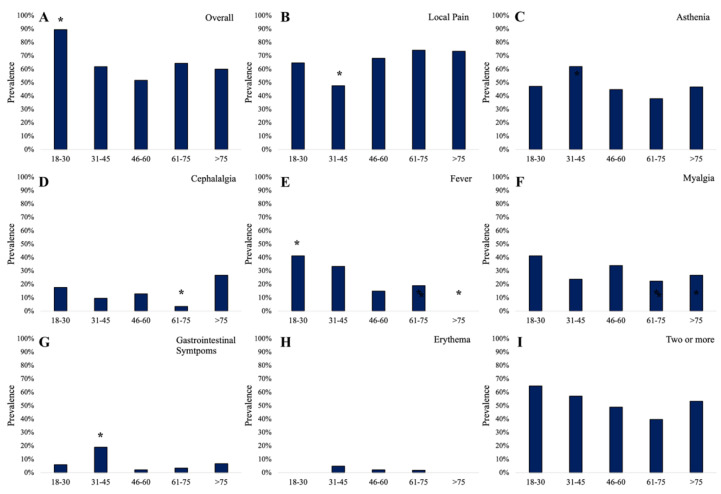
The overall prevalence of adverse events (**A**) and the prevalence of local pain (**B**), asthenia (**C**), cephalalgia (**D**), fever (**E**), myalgia (**F**), gastrointestinal symptoms (**G**), erythema (**H**), and the prevalence of two or more AEs (**I**) in vaccinated participants according to group of age; * statistically significant data.

The number of AEs was inversely correlated with age (Spearman, r= −0.15; *p* = 0.03). In Figure 4, the prevalence of AEs according to groups of age is reported. Overall, AEs showed higher prevalence in the group “18–30 years” (89.5%) compared to other groups (χ^2^= 6.9; phi= 0.163; OR = 5.9 [95% CI: 1.33 to 26.15]; *p* = 0.009) (Figure 4A). Patients with age comprised between 31 and 45 years were at low risk of local pain (χ^2^= 4.47; phi= −0.168; OR = 0.38 [95% CI: 0.15 to 0.95]; *p* = 0.034) (Figure 4B), while they were at higher risk of gastrointestinal symptoms (χ^2^= 8.04; phi= 0.226; OR = 6.21 [95% CI: 1.51 to 25.4]; *p* = 0.019) (Figure 4G). Cephalalgia was reported less in the group of age “61–75 years” (χ^2^= 5.1; phi= −0.18; OR = 0.2 [95% CI: 0.04 to 0.92]; *p* = 0.024) (Figure 4D). Fever was reported by 41.2% of patients in the group of age “18–30 years” (χ^2^= 5.16; phi= 0.181; OR = 3.25 [95% CI: 1.12 to 9.36]; *p* = 0.048) and it was absent in the group “>75 years” (χ^2^= 4.2; phi= −0.163; OR = 0.78 [95% CI: 0.71 to 0.85]; *p* = 0.042) (Figure 4E).

AEs showed lower prevalence in patients with inflammatory neuropathies compared to other groups (χ^2^= 8.5; phi= −0.181; OR = 0.46 (95% CI: 0.27 to 0.78); *p* = 0.004), while patients with toxic neuropathy reported the higher prevalence of AEs (83.3%) but this did not reach the statistical significance (*p* = 0.12) (Table 2). Asthenia was more common in hereditary neuropathies (χ^2^ = 6.17; phi = 0.198; OR = 2.23 (95% CI: 1.18–4.24); *p* = 0.013), while no other associations were found between AEs and neuropathy subtypes (all *p* > 0.05; Table 2). AEs were slightly common among patients who received viral-vector-based vaccines compared to those who received mRNA-based vaccines (77.8% vs. 60.6%; OR = 2.72 (95% CI: 0.46–11.16); Fisher’s exact test: *p* = 0.49). Patients with at least an AE received a higher median number of doses compared to those without AE (3 (2–3) vs. 2 (2–3); *p* = 0.001). No serious AEs and life-threatening conditions were observed.

### 3.3. Medications and Adverse Events

All the medications are reported in Table 1. AEs occurred similarly in patients who were taking medications during vaccination (*n* = 93/147, 58.9%) and in those who were not taking any (*n* = 65/111, 41.1%; *p* = 0.44). Number of medications was significantly higher in patients with toxic and deficiency neuropathies compared to others (*p* = 0.001; Table 1). Steroids were associated with a lower risk of developing AEs (18.8% vs. 64%; χ^2^= 12.97; phi = −0.224; OR = 0.13 [95% CI: 0.036–0.47]; *p* < 0.0001). As well, patients who were taking immunosuppressants developed fewer AEs compared to those who were not taking these drugs (30% vs. 62.2%: OR = 0.257 [95% CI: 0.065–1.19]; Fisher’s exact test: *p* = 0.05). Prevalence of AEs was higher in patients who were taking analgesics (88.9% vs. 59.2%; χ^2^ = 6.23; phi = 0.155; *p* = 0.013), as well as in patients who were taking nutraceutics (76.9% vs. 58.4; χ^2^ = 4.76; phi = 0.136; *p* = 0.029). No associations emerged while considering other medications (all *p* > 0.05). There was a little inverse correlation between number of medications and number of AEs, but this did not reach the statistical significance (Spearman, r= −0.056; *p* = 0.3).

### 3.4. Previous COVID-19 and Reactogenicity to Vaccination

In total, 24 participants experienced COVID-19 before vaccination (Table 1). Reactogenicity to vaccination was higher in people who experienced COVID-19 before vaccination compared with those who experienced COVID-19 after vaccination or with those who did not experience any COVID-19 after vaccination (*n* = 18/24, 75% vs. *n* = 140/234, 59.8%; OR = 2 [95% CI: 0.77–5.26]), but these data did not reach the statistical significance (*p* = 0.14).

### 3.5. Patient’s Reported Outcomes

After vaccination, 223 patients (86.4%) reported unchanged or improved symptoms related to neuropathy, and 35 (13.6%) patients reported a worsening, respectively (Table 3). Subjective reported outcome change was not associated with gender, medications, and type of vaccine received (all *p* > 0.05), while an association was found with COVID-19 before vaccination (OR = 3.03 [1.15–7.95]; Fisher’s extract test: *p* = 0.03), the occurrence of AEs (χ^2^ = 6; phi = 0.153; *p* = 0.014), and age comprised between 18 and 30 years old (OR = 3.34 [1.18–9.48]; Fisher’s extract test: *p* = 0.03) (Table 3).

## 4. Discussion

In this study, we investigated the rate of adherence to COVID-19 vaccination and local and systemic reactogenicity to vaccination in a cohort of 268 patients affected by polyneuropathy (Table 1). The level of adherence to the Italian COVID-19 vaccination program was very high, accounting for about 96% of respondents. However, VH was not completely disrupted. In particular, the adherence rate to vaccination was higher in patients with hereditary neuropathy compared to others, while we found a lower adherence rate in patients with anti-MAG neuropathy (66.7%; Table 1). These data are according to existing evidence. Indeed, many patients affected by neurological diseases, especially autoimmune, are afraid to receive vaccination due to a self-perception of a pre-existing medical condition contraindicated with vaccination [16]. Of note, Holtz et al. showed a very low attitude toward vaccination after GBS [17]. Of note, as vaccines may stimulate the immune response, it is reasonable to assume that reactogenicity and symptom worsening following vaccination might be more common in inflammatory neuropathies if compared to hereditary neuropathies because the former are characterized by elevated systemic inflammation activity, while neurodegeneration is predominant in the latter [18,19,20]. Among the general population, the most common AE following BNT162b2 is local pain (66–83%), followed by fatigue (51–59%), cephalalgia (25–52%), myalgia (19–37%), and gastrointestinal symptoms (8–12%) [21], with a higher prevalence in younger people. These symptoms are similar compared to those occurring after mRNA-1273 administration [22]. After ChAdOx1 nCoV-19 administration a higher prevalence of fatigue was noted (70%), together with myalgia (60%) and fever (18%) [23]. When compared with the aforementioned data, results from our study show a lower prevalence of AEs in patients with neuropathies compared to the general population. Furthermore, we showed that having an inflammatory neuropathy is associated with lower odds of AEs following vaccination (OR= 0.46; Table 2) and it was not associated with a reported worsening (Table 3). In our study, about 81% of patients with inflammatory neuropathy were CIDP; thus, our results do not support a higher prevalence of AEs in these patients. In contrast, patients with inflammatory neuropathies are at higher risk of severe COVID-19 [14]; hence, based on our results, we would encourage these patients to receive vaccination. However, we showed steroids and immunosuppressants are associated with lower odds of AEs. Thus, the lower prevalence of AEs in the group of inflammatory neuropathies compared to others might be related to steroids and immunosuppressants consumption. Indeed, it has been demonstrated that premedication may reduce the AE occurrence after vaccination [23]. Moreover, although only 3.5% of participants and the 6.4% with inflammatory neuropathies received the viral-vectors-based vaccine in our cohort, our results go straight forward to the safety of viral-vectors-based vaccines in these patients according to the previous report on other autoimmune neurological diseases, such as multiple sclerosis and myasthenia gravis [24,25,26]. Overall, we found that younger patients and particularly those in the age comprised between 18 and 30 years old, as well as females and those having had COVID-19 before vaccination were associated with higher odds of AEs (Table 2). These data are strongly reported in the literature [27,28]. Therefore, it seems that reactogenicity following vaccination was driven principally by gender, COVID-19 before vaccination, and younger age rather than the underlying neuropathy (Table 1 and Table 2). Moreover, having had COVID-19 before vaccination is a well-known risk factor for developing higher AEs following vaccination [29]. The higher reactogenicity following vaccination in females could be explained by the higher immune systemic response to non-self-antigen (e.g., spike protein); indeed, women are more prone to developing autoimmune disorders [30]. A total of 35 patients (13%) reported a symptom worsening following vaccination, which is lower than reported by Vivekanandam et al. in a cohort of patients with channelopathies (38%) [31]. Finally, an association between the occurrence of AEs (asthenia, fever, and myalgia; Table 3), having had COVID-19 before vaccination, as well as age comprised between 18 and 30 were factors associated with higher odds of perceived worsening of the neuropathy-related symptoms after vaccination (Table 3). Considering that having had COVID-19 before vaccination led to both higher prevalence of AEs and perceived symptom worsening, whether testing for COVID-19 is necessary before giving vaccination in patients with neuropathy may be explored in future studies. This study has several limitations. First, given the retrospective design, the main trouble may be the information bias due to patients’ forgetfulness. Furthermore, the use of a web-based questionnaire might have underestimated data collection because of self-compilation or data entry errors. Moreover, we explored the transient reactogenicity that occurred after vaccination and lasted less than a week; therefore, medium- and long-term vaccine-induced AEs were not evaluated. Future studies are needed to assess the medium- and long-term AEs after vaccination in patients with polyneuropathies. In our cohort, many participants received BNT162b2 and, consequently, the AEs related to mRNA-1273 and viral-vectors-based vaccines may be underestimated. Finally, as third-level Neuromuscular Clinical Centers, we enrolled many patients with inflammatory or hereditary neuropathy; thereby, results may not be sound for patients with endocrine, toxic, or deficiency neuropathies. However, the web-based cross-sectional design represents a preliminary, less expensive, and “social distancing” approach to investigate the adherence to vaccination in vulnerable patients (i.e., patients with neuropathy), as well as the prevalence of vaccine-related AEs. Thus, we think our results may drive both neurologists and patients with neuropathy toward vaccination. Further prospective studies are needed to explore the long-term safety of vaccination in these patients.

## 5. Conclusions

Vaccination against SARS-CoV-2 reached a higher rate of adherence in a cohort of patients affected by polyneuropathy, although VH was still elevated in patients with anti-MAG neuropathy. Patients with neuropathies showed an AE prevalence comparable to the general population, with a lower prevalence in patients with inflammatory subtype, probably due to immunosuppressants assumption. Being female, having had COVID-19, and younger age were associated with higher odds of AEs, while having had COVID-19 before vaccination and having had an AE were associated with higher odds of perceived symptom worsening after vaccination. As vaccines against-SARS-CoV-2 showed a good short-term safety profile in patients with inflammatory, hereditary, diabetic, toxic, and deficiency neuropathies, we encourage vaccination in these patients.

## Figures and Tables

**Table 1:** Demographic and clinical features of the participants included in the final analysis.

	Inflammatory	Hereditary	Diabetic	Toxic	Deficiency	Overall
No. of participants, *n*, (%)	99, (36.9)	109, (40.5)	32, (11.9)	13, (4.8)	15, (5.6)	268
Female, *n*, (%)	28, (28.3)	57, (52.3)	13, (40.6)	3, (23.1)	11, (73.3)	105, (39.2)
Age, median (IQR), y	59 (50–67)	56 (43–67)	67 (58–75)	62 (51–69)	60 (57–66)	59 (48–68)
Adherence, *n*, (%)	93, (93.9)	109, (100)	30, (93.8)	12, (92.3)	14, (93.3)	258, (96.3)
BNT162b2	77, (82.8)	97, (89)	23, (76.7)	11, (91.7)	11, (78.6)	219, (84.9)
mRNA-1273	10, (10.8)	9, (8.3)	7, (23.3)	1, (8.3)	3, (21.4)	30, (11.6)
Viral vectors-based	6, (6.4)	3, (2.7)	0	0	0	9, (3.5)
No. of doses, mean ± SD	2.41 ± 0.53	2.50 ± 0.58	2.33 ± 0.48	2.42 ± 0.67	2.43 ± 0.51	2.44 ± 0.57
COVID before vaccination, *n*, (%)	8/93 (8.6)	11/109 (10)	4/32 (12.5)	0	1/14 (7.1)	24/258 (9.3)
Asymptomatic	2, (25)	3, (27.3)	1, (25)		0	6, (25)
Flu-like symptoms	2, (25)	6, (54.5)	3, (75)		0	11, (45.8)
Hospitalized	4, (50)	2, (18.2)	0		1, (100)	7, (29.2)
Medications, *n*, (%)	69/93, (74.2)	48/109, (44)	13/30, (43.3)	11/12, (91.7)	6/14, (42.9)	147/258, (57)
Steroids	14, (20.3)	0	0	1, (9)	1, (16.7)	16, (10.9)
Immunosuppressants	7, (10.1)	0	1, (7.7)	1, (9)	1, (16.7)	10, (6.8)
Immunoglobulins	38, (55)	0	0	0	0	38, (25.9)
Analgesics	6, (8.7)	8, (16.6)	3, (23)	1, (9)	0	18, (12.2)
Antidepressants	5, (7.2)	5, (10.4)	0	1, (9)	1, (16.7)	13, (8.1)
Antiepileptic drugs	14/69, (20.3)	9, (18.8)	9, (69.2)	9/11, (81.8)	1, (16.7)	42, (29.3)
Patisiran	0	18, (37.5)	0	0	0	18, (12.2)
Nutraceutics	5/69, (7.2)	18/48, (37.5)	6/13, (46.2)	6/11, (54.5)	4/6, (66.6)	39, (26.5)
No. medication, mean ± SD	1.31 ± 0.55	1.46 ± 0.8	1.77 ± 1.16	2.27 ± 0.9	1.83 ± 0.4	1.53 ± 0.84

**Table 2:** Prevalence of AEs in vaccinated patients according to neuropathy subtype.

	Inflammatory *N* = 94	Hereditary *N* = 109	Diabetic *N* = 30	Toxic *N* = 12	Deficiency *N* = 14	Overall *N* = 258
AEs prevalence *n*, (%)	46, (48.9)	74, (67.9)	18, (60)	10, (83.3)	10, (71.4)	158, (61.2)
Females	20, (43.5)	41, (55.4)	9, (50)	3, (30)	2, (20)	75/102, (73.5)
Males	26, (56.5)	33, (44.6)	9, (50)	7, (70)	8 (80)	83/156, (53.2)
Age, median (IQR), y	61 (54–67)	53 (38–68)	67 (57–79)	62 (43–66)	63 (57–71)	60 (47–69)
AEs type *n*, (%)						
Local pain	33, (71.7)	47, (63.5)	10, (55.6)	8, (80)	9, (90)	107, (67.7)
Asthenia	16, (34.8)	41, (55.4)	6, (8.5)	4, (40)	4, (40)	71, (44.9)
Cephalalgia	2, (4.3)	11, (14.9)	4, (22.2)	0	0	17, (18.8)
Fever	11, (23.9)	14, (18.9)	5, (27.8)	1, (10)	1, (10)	32, (20.2)
Myalgia	17, (37)	24, (32.4)	2, (11.1)	1, (10)	1, (10)	45, (28.5)
Gastrointestinal	4, (8.7)	2, (2.7)	2, (11.1)	0	1, (10)	9, (5.7)
Erythema	2, (4.3)	1, (1.4)	0	0	0	3, (1.9)
Two or more	22, (47.8)	40, (54.1)	9, (50)	3, (30)	3, (30)	77, (48.7)
No. of AEs, mean ± SD	1.85 ± 1.1	1.89 ± 1	1.61 ± 0.7	1.4 ± 0.5	1.6 ± 1.16	1.8 ± 1

**Table 3:** Comparison between demographic and vaccination features between stable or improved and worsened patients.

	Stable or Improved *N* = 223	Worsened *N* = 35	Odd Ratio (CI 95%)	*p*
Females *n*, (%)	87 (38.8)	16 (45.7)	0.83 (0.33–2)	0.4
Age, median (IQR), y	60 (50–69)	56 (42–65)		0.08
Group of age *n*, (%)				
18–30 years	13, (5.8)	6, (17.1)	3.34 (1.18–9.48)	0.03
31–45 years	31, (13.9)	3, (8.6)	0.58 (0.17–2)	0.59
46–60 years	77, (34.4)	14, (40)	1.3 (0.62–2.68)	0.49
61–75 years	81, (36.2)	9, (10)	1.5 (0.27–1.36)	0.2
>75 years	22, (9.8)	3, (8.6)	3.3 (1.18–9.48)	0.81
COVID before vaccination *n*, (%)	17, (7.6)	7/35 (20)	3 [1.15–7.95]	0.03
Medications *n*, (%)	126 (56.5)	21 (60)	1.1 (0.56–2.4)	0.7
Viral vectors-based vaccine *n*, (%)	7, (3.1)	2, (5.7)		0.46
mRNA-based vaccine *n*, (%)	216 (96.9)	33 (94.3)		0.46
Adverse events *n*, (%)	130/223 (58.3)	28/35 (80)	2.9 (1.11–6.83)	0.014
Local pain	92, (70.8)	15, (53.6)	0.47 (0.2–1.1)	0.08
Asthenia	52, (40)	19 (67.9)	3.2 (1.33–7.53)	0.007
Cephalalgia	11, (8.5)	6, (21.4)	2.9 (0.98–8.8)	0.08
Fever	22, (16.9)	10, (35.7)	2.7 (1.11–6.7)	0.03
Myalgia	30, (23.1)	15, (53.6)	3.8 (1.65–8.97)	0.001
Gastrointestinal	6, (4.6)	3, (10.7)	2.48 (0.58–10.58)	0.2
Erythema	3, (2.3)	0		0.27
Two or more	56, (43.1)	21, (75)	3.96 (1.58–9.98)	0.002
Polyneuropathy subtype *n*, (%)				
Inflammatory	82, (36.8)	11, (31.4)	0.79 (0.37–1.7)	0.5
Hereditary	94, (42)	15, (42.9)	1 (0.5–2.11)	0.9
Diabetic	27, (12.1)	3, (8.6)	0.68 (0.19–2.37)	0.6
Toxic	8, (3.6)	4, (11.4)	3.5 (0.99–12.2)	0.06
Deficiency	12, (5.4)	2, (5.7)	1 (0.23–4.98)	1

## Data Availability

Data are available from the corresponding author upon a reasonable request.

## Supplementary Materials

The following supporting information can be downloaded at: https://www.mdpi.com/article/10.3390/brainsci12101396/s1, File S1: Questionnaire; File S2: Supplementary analyses.
