# Supplementary material for: Adherence and Reactogenicity to Vaccines against SARS-COV-2 in 285 Patients with Neuropathy: A Multicentric Study
Source: Brain Sci. 2022 Oct 16;12(10):1396. doi: 10.3390/brainsci12101396 (PMC9599423; doi:10.3390/brainsci12101396)
Supplement: Supplementary file 1 [file brainsci-12-01396-s001.zip › Supplementary File S1.pdf]

Studio osservazionale sulla aderenza alla vaccinazione contro SARS-CoV-2 in pazienti affetti da neuropatia e raccolta degli eventi avversi correlati al vaccino.

Ambulatorio Malattie Neuromuscolari rare
AOUP Paolo Giaccone, Università degli Studi di Palermo

Il seguente questionario è raccolto in forma anonima, quindi in nessuna occasione dovrà scrivere il suo nome e/o cognome.

---

*Campo obbligatorio

1. Il presente questionario ha lo scopo di raccogliere informazioni sulla aderenza o meno alla vaccinazione contro SARS-CoV-2 in soggetti affetti da neuropatia e di età maggiore o uguale a 18 anni. Inoltre, verranno raccolte informazioni demografiche come sesso ed età, la terapia praticata ed eventuali eventi avversi correlati alla vaccinazione. L' obiettivo del presente studio è di quantificare l'aderenza alla vaccinazione in soggetti con neuropatia e di studiare gli eventi avversi correlati al vaccini in questa categoria di pazienti. I dati verranno raccolti e utilizzati in forma anonima. Presta il suo consenso a partecipare allo studio? La risposta "no" terminerà in maniera automatica il questionario e la raccolta dei dati. *

   Contrassegna solo un ovale.

   - Sì
   - No (in tal caso il questionario termina qui!)

2. Sesso *

   Contrassegna solo un ovale.

   - Uomo
   - Donna

3. Età *

4. Fascia d'età *

Contrassegna solo un ovale.

- 18-30
- 31-45
- 46-60
- 61-75
- >75

5. Dove è seguito per la sua neuropatia? *

Contrassegna solo un ovale.

- Palermo
- Napoli
- Chieti
- Bari
- Salerno
- Altro: ____________

6. Qual è la sua diagnosi? *

Contrassegna solo un ovale.

- ( ) Neuropatia diabetica
- ( ) CIDP (polineuropatia infiammatoria demielinizzante cronica)
- ( ) amiloidosi ereditaria
- ( ) neuropatia ereditaria (CMT)
- ( ) neuropatia carenziale
- ( ) neuropatia tossica da farmaci (chemioterapici o altro tipo)
- ( ) neuropatia anti-MAG
- ( ) Neuropatia ereditaria (HNPP)
- ( ) Altro: ______________________

7. Ha contratto il COVID-19? *

Contrassegna solo un ovale.

- ( ) si, prima di ricevere il vaccino
- ( ) si, dopo aver ricevuto il vaccino
- ( ) no *Passa alla domanda 9.*

8. Se ha contratto il COVID-19, può specificare che forma ha avuto? *

Contrassegna solo un ovale.

- ( ) una forma asintomatica
- ( ) sintomi lievi (alterazioni del gusto o olfatto, febbre)
- ( ) una forma con polmonite con ospedalizzazione
- ( ) una forma grave con accesso in rianimazione

INFORMAZIONI SULLA VACCINAZIONE

9. Ha ricevuto il vaccino anti-COVID19? *

Contrassegna solo un ovale.

- Sì
- no

10. Quale tipo di vaccino? *

Contrassegna solo un ovale.

- Pfizer
- Moderna
- Astrazeneca
- J&J

11. Scriva la data della prima dose *

____

12. Scriva la data della seconda dose *

____

13. Scriva la data della terza dose *

____

14. Quali effetti collaterali correlati alla vaccinazione ha avuto dei seguenti (presentati in un periodo di tempo che va dalla vaccinazione fino a 7 giorni) ? *

Seleziona tutte le voci applicabili.

- [ ] Dolore locale al sito di inoculo
- [ ] Spossatezza/stanchezza (astenia)
- [ ] Febbre (temperatura superiore a 37.2°C)
- [ ] Dolori muscolari (mialgia)
- [ ] Sintomi gastrointestinali (nausea, vomito, diarrea, dolori addominali)
- [ ] Cefalea
- [ ] Eritema (rash cutaneo)

15. Quali farmaci assumeva nel periodo in cui è stato sottoposto alla vaccinazione? *

Contrassegna solo un ovale.

- ◯ Analgesici
- ◯ Patisiran
- ◯ Corticosteroide
- ◯ Nutraceutici (integratori vitaminici, L-acetil-carnitina, acido alfa-lipoico, etc)
- ◯ Antidepressivi (amitriptilina, duloxetina, venlafaxina, escitalopram, etc)
- ◯ Antiepilettici (valproato, gabapentin, pregabalin, etc)
- ◯ Immunoglobuline

16. Quanti farmaci di quelli sopraelencati sta assumendo? *

____

17. Valutazione soggettiva sulla modificazioni dei sintomi dovuti alla neuropatia nel periodo successivo la vaccinazione. Indichi la sua valutazione sui propri sintomi. *

Contrassegna solo un ovale.

- Rimasti immodificati
- Sono peggiorati
- Sono migliorati

Questi contenuti non sono creati né avallati da Google.

Google Moduli
