# Supplementary material for: Adherence and Reactogenicity to Vaccines against SARS-COV-2 in 285 Patients with Neuropathy: A Multicentric Study
Source: Brain Sci. 2022 Oct 16;12(10):1396. doi: 10.3390/brainsci12101396 (PMC9599423; doi:10.3390/brainsci12101396)
Supplement: Supplementary file 1 [file brainsci-12-01396-s001.zip › Supplementary File S2.pdf]

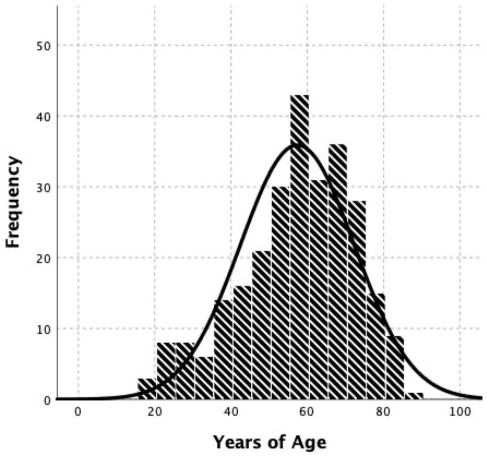

Figure S2.1 Kolmogorov-Smirnov Test assessing the distribution of “Age”

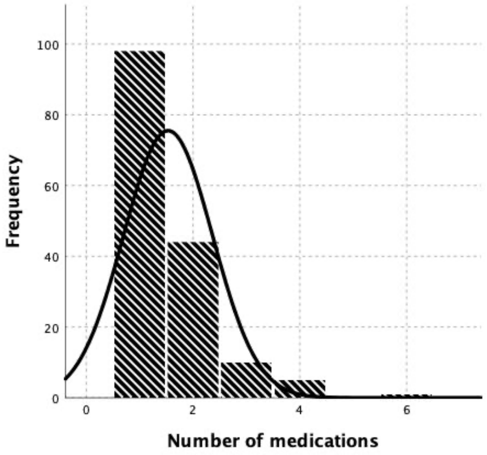

Figure S2.2 Kolmogorov-Smirnov Test assessing the distribution of “Number of medications”

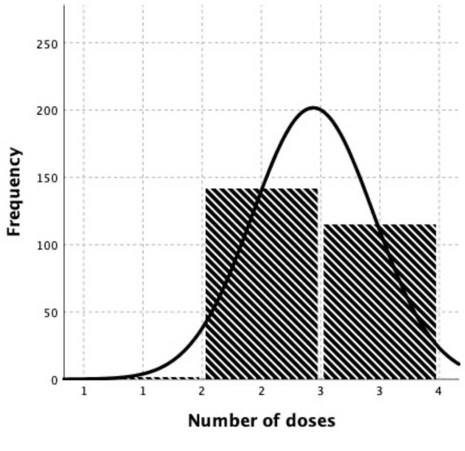

Figure S2.3 Kolmogorov-Smirnov Test assessing the distribution of “Number of doses”

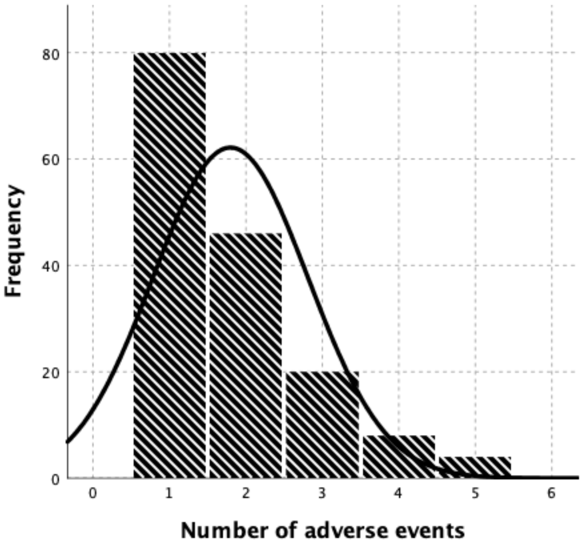

Figure S2.4 Kolmogorov-Smirnov Test assessing the distribution of “Number of adverse events”

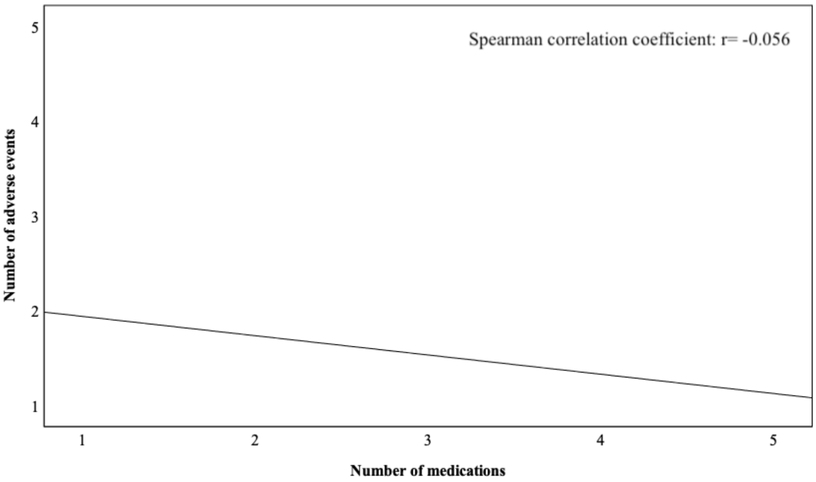

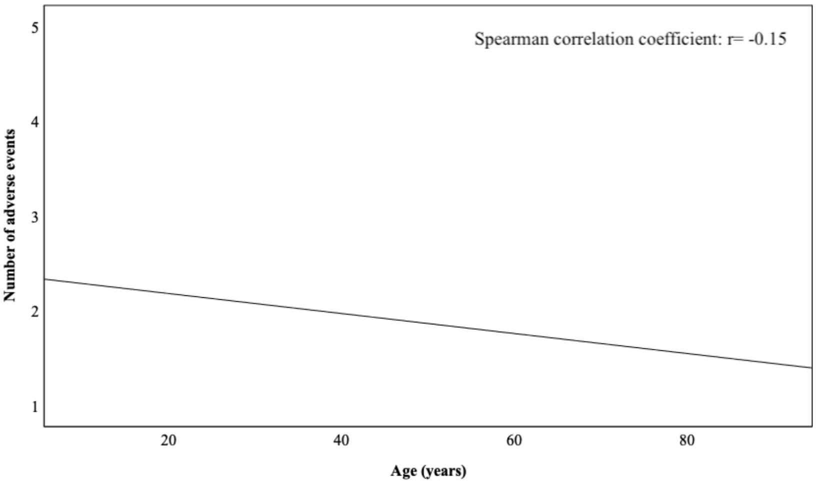

Figure S2.5 Spearman’s graphs of the correlation between number of adverse events and number of medications (upper graphs) and between number of adverse events and years of age (lower graph).
